# A Course of Practical Histology

**Published:** 1877-03

**Authors:** 


A COURSE OF PRACTICAL HISTOLOGY—BEING AN INTRODUCTION TO THE USE OF THE MICROSCOPE. By Edward Albert Schaffer, Assistant Professor of Physiology, in University College, London; with illustrations on wood; 279 pages. Publishers, Henry C. Lea, Philadelphia. 1877.

